# Bayesian Phylogeography and Pathogenic Characterization of Smallpox Based on *HA*, *ATI*, and *CrmB* Genes

**DOI:** 10.1093/molbev/msy153

**Published:** 2018-08-07

**Authors:** Dillon C Adam, Matthew Scotch, Chandini Raina MacIntyre

**Affiliations:** 1 Biosecurity Program, Kirby Institute, Faculty of Medicine, University of New South Wales, Sydney, NSW, Australia; 2 School of Public Health and Community Medicine, University of New South Wales, Sydney, NSW; 3 Biodesign Center for Environmental Health Engineering, Biodesign Institute, Arizona State University, Tempe, AZ; 4 Department of Biomedical Informatics, College of Health Solutions, Arizona State University, Tempe, AZ; 5 College of Public Service and Community Solutions and College of Health Solutions, Arizona State University, Tempe, AZ

**Keywords:** Variola virus, smallpox, phylogeography, epidemics, public health, biosecurity

## Abstract

*Variola virus* is at risk of re-emergence either through accidental release, bioterrorism, or synthetic biology. The use of phylogenetics and phylogeography to support epidemic field response is expected to grow as sequencing technology becomes miniaturized, cheap, and ubiquitous. In this study, we aimed to explore the use of common VARV diagnostic targets hemagglutinin (*HA*), cytokine response modifier B (*CrmB*), and A-type inclusion protein (*ATI*) for phylogenetic characterization as well as the representativeness of modelling strategies in phylogeography to support epidemic response should smallpox re-emerge. We used Bayesian discrete-trait phylogeography using the most complete data set currently available of whole genome (*n* = 51) and partially sequenced (*n* = 20) VARV isolates. We show that multilocus models combining *HA, ATI*, and *CrmB* genes may represent a useful heuristic to differentiate between VARV Major and subclades of VARV Minor which have been associated with variable case-fatality rates. Where whole genome sequencing is unavailable, phylogeography models of *HA, ATI*, and *CrmB* may provide preliminary but uncertain estimates of transmission, while supplementing whole genome models with additional isolates sequenced only for *HA* can improve sample representativeness, maintaining similar support for transmission relative to whole genome models. We have also provided empirical evidence delineating historic international VARV transmission using phylogeography. Due to the persistent threat of re-emergence, our results provide important research for smallpox epidemic preparedness in the posteradication era as recommended by the World Health Organisation.

## Introduction

*Variola virus* (VARV), is a large (∼186 kb), linear, double-stranded DNA virus of the *Orthopoxvirus* (OPV) genus (King 2012), and the etiological agent of smallpox (Ledingham 1931). Smallpox is considered a disease of antiquity, however, its use as a bioterrorism agent has been debated for decades (Henderson and Arita 2014). In 2017, scientists successfully completed the de novo synthesis of a poxvirus believed to be no-longer circulating in nature (Tulman et al. 2006; Noyce et al. 2018), prompting fears of potential smallpox re-emergence through advances in synthetic biology (Koblentz 2017). Currently, there are 571 known VARV samples in two WHO authorized Collaborating Centres in the United States and the Russian Federation (Alcami et al. 2010). Despite strict regulations surrounding VARV research, insider threat, accidental release, and unauthorized experimentation and storage of potential agents of bioterrorism are not without precedent (MacIntyre and Engells 2016; Pillai 2016). Furthermore, the threat of synthetic biology means regulated containment or the destruction of remaining samples no longer represents a barrier or failsafe against future outbreaks (Damon et al. 2014; MacIntyre 2015; Mitchell and Ellis 2017). A recent study modelling the impact of re-emergent smallpox has shown widespread infection among immunologically naïve persons as vaccination for VARV declined when eradication progressed (MacIntyre et al. 2018). However, the highest mortality would occur in those aged > 45 years as vaccine induced immunity wains concurrent with unprecedented levels of iatrogenic and infectious disease-associated immunosuppression in contemporary society (MacIntyre et al. 2018). As modern molecular methods and tools become more readily accessible and affordable, the threat posed by re-emergent smallpox cannot be ignored.

Prior to official eradication in 1980 (Behbehani 1983), VARV existed as two primary clades reasonably associated with differing mortality: high-mortality “major” (>10% CFR) and low-mortality “minor” known as alastrim (<1% CFR) (Massung et al. 1995, 1996). Records are suggestive of a third clade common to West Africa in the late 20th century associated with intermediate mortality (1–10% CFR) (Shaffa 1972; Fenner et al. 1988), and efforts were made in the late 1960s to classify VARV into three taxonomic subspecies (Dumbell et al. 1961). Whole genome phylogenetic studies correlating VARV isolates with aggregate country CFR further support the existence of a third clade common to Western Africa associated with intermediate mortality, however, there remains some phylogenetic disagreement between CFR within VARV major, particularly isolates from East Africa (Esposito et al. 2006). Diagnosis through laboratory confirmation requires the detection of VARV DNA using polymerase chain reaction (PCR). Common VARV specific nucleic acids targets include the hemagglutinin (*HA)*, cytokine response modifier B (*CrmB*) and A-type inclusion protein (*ATI*), and can differentiate VARV from other OPV such as monkeypox and camelpox (Ropp et al. 1995; Meyer et al. 1997; Hansen et al. 2009; Kurth and Nitsche 2011; Okeke et al. 2012, 2014). The potential for VARV differentiation by clade using reduced data sets including *HA, ATI*, or *CrmB* has yet to be explored, but could assist with future preparedness planning and outbreak response due to approximate associations by clade with CFR and therefore approximations of innate-viral pathogenesis (Esposito et al. 2006; Li et al. 2007). Understanding the use of genes like *HA* as a potential heuristic approximation to whole genome (WG) phylogeography may also be useful to support rapid real-time outbreak response in the field should smallpox re-emerge.

In this study, we aim to use Bayesian phylogenetic methods to explore the utility for characterization of VARV based on diagnostic genes *HA*, *ATI*, and *CrmB* as well as explore the phylogeographic signal available to each individual gene and in combination. We also aim to describe the phylogeography of smallpox using all currently sequenced whole VARV genomes and investigate representative biases by supplementing WG models with additional isolates sequenced for *HA* only.

## Results

**Fig. 1.**
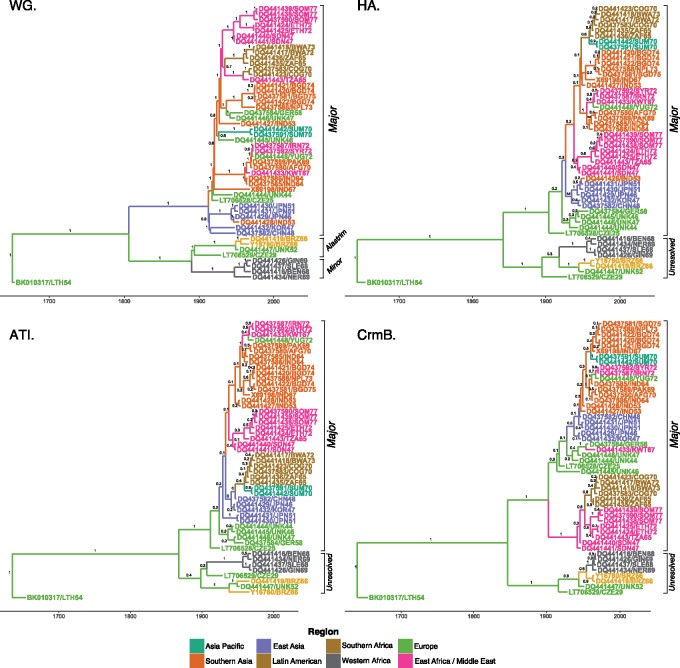
Time-rooted phylogenetic characterization of whole genome and single-locus models of *HA*, *ATI* and *CrmB* genes using identical taxonomic data sets (*n* = 51). Values on ancestral nodes represent posterior probabilities. Tip are colored by sampling region and edges colored by inferred ancestral origin.

Maximum Clade Credibility (MCC) trees using reduced data sets of *HA*, *ATI*, or *CrmB* demonstrated various degrees of incongruence compared with the WG model. Minor subclades could not be differentiated in any single gene model of *HA*, *ATI*, or *CrmB* (fig. 1). The topology of taxa within clades also varied across all single models compared with the WG. Temporally and spatially related taxa grouped together yet the inferred ancestral relationships between groups of taxa differed between models. The posterior probability at most ancestral nodes was also reduced in each model when compared with WG models.

**Fig. 2.**
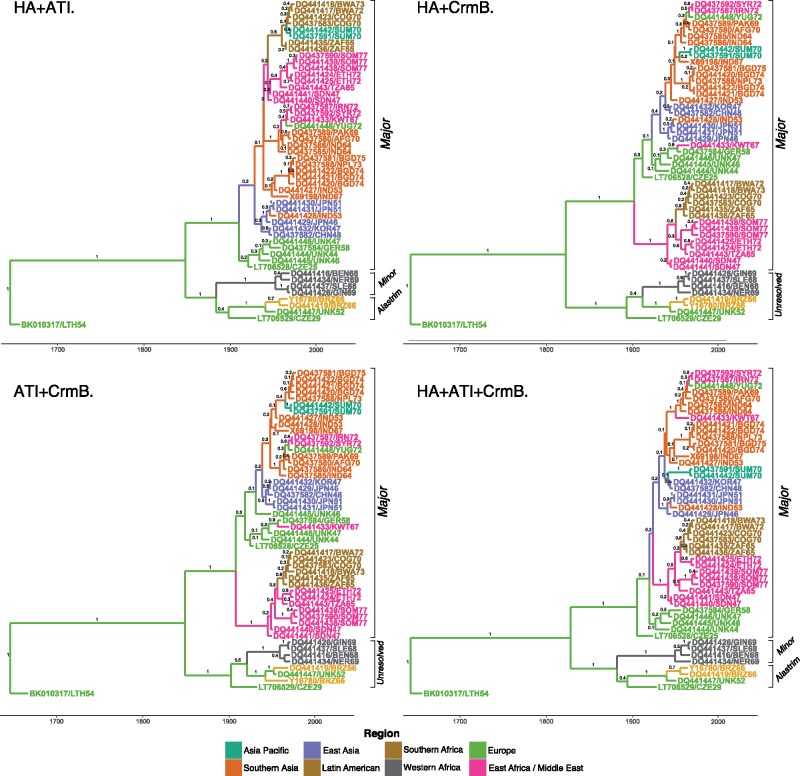
Time-rooted phylogenetic characterization of multilocus models of *HA*, *ATI* and *CrmB* genes using identical taxonomic data sets (*n* = 51) extracted from isolates sequenced as WG. Values on ancestral nodes represent posterior probabilities. Tip are colored by sampling region and edges colored by inferred ancestral origin using a Bayesian Stochastic Search Variable Selection framework.

**Fig. 3.**
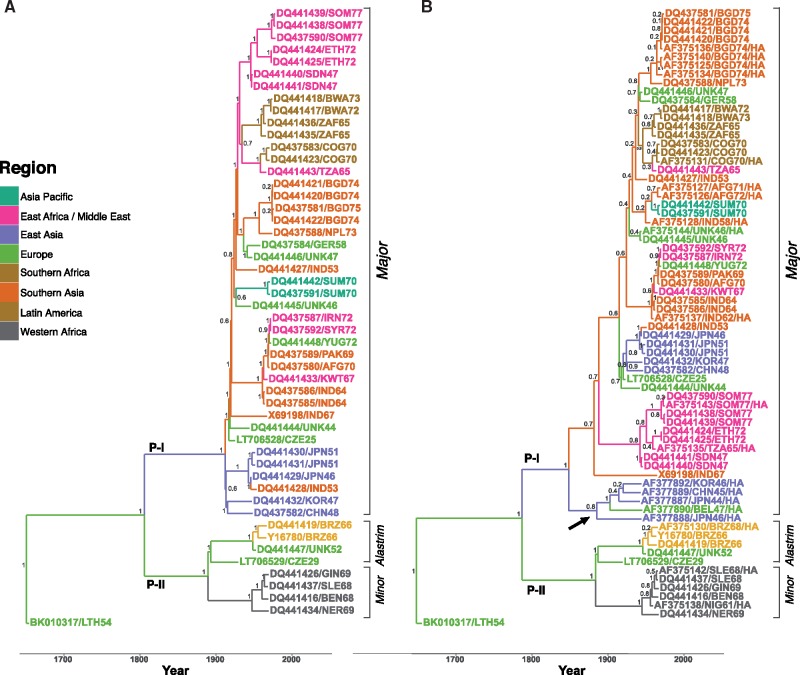
Time-rooted phylogenetic and phylogeographic characterization of VARV isolates. Values on ancestral nodes represent posterior probabilities. Tip names are colored by sampling region and edges colored by region state. (*A*) Fifty-one WG VARV isolates. (*B*) Fifty-one WG VARV isolates aligned with 20 additional isolates sequenced for *HA* only. *HA*-only taxa are indicated with “/HA” suffix. A single monophyletic clade of *HA*-only taxa of East Asian/European origin is indicated with an arrow.

Only multilocus models combining two (*HA* and *ATI*) or three (*HA*, *ATI*, and *CrmB*) loci were able to differentiate between subclades (fig. 2), however in both final MCC trees, the monophyly of alastrim had low posterior support (0.4). The topology also varied within the minor clades of the multilocus *HA* and *ATI* model compared with the WG model. Only models combining all three loci (*HA, ATI*, and *CrmB)* generated sufficient statistical support to differentiate both minor subclades as well as the topology within each subclade as seen in the WG model, but the alastrim monophyly again had low posterior support (0.4). While topologies within the VARV Major clade varied between all models compared with the WG, in no instance was taxa characterized as VARV major in the WG model allocated to either minor subclade in the MCC tree of the reduced data set models. In WG models supplemented with additional isolates sequenced for *HA* only, taxa were characterized alongside spatially and temporally related WG taxa yet the posterior probability was reduced (fig. 3*B*). In the final MCC tree, a monophyletic clade of *HA-*only isolates sampled between 1944 and 1946 from East Asia and Europe is separate from all other East Asian and European clusters with related sampling times (posterior probability 0.8**)**. In final MCC tree using *HA* only sequences extracted from WG isolates and supplemented with additional *HA* isolates, this cluster remains (supplementary fig. S1*B*, Supplementary Material online).

Phylogeography analysis of all VARV taxa sequenced and available as whole genomes (*n* = 51) reveal ten statistically supported (BF > 3) routes of transmission between eight discrete regions (56 possible asymmetric routes). These are ranked by strength of support in table 1. The highest support for transmission (considered decisive as BF > 100) occurs between Southern Asia to the East Africa and Middle East (BF = 4703.65) and the lowest (supported as 10 > BF > 3) from Western Africa to East Asia (BF = 3.48). Single and multilocus models of *HA, ATI*, and *CrmB*, supported the top six statistically supported routes from the WG model with variable strength and are also ranked in table 1. Rankings were discordant between the reduced data set models and the WG model. Route Southern Asia to Asia Pacific was supported (10 > BF > 3) only in WG models (BF = 8.37) and all models incorporating *CrmB* with variable support (BF = 6.59–24.15; strong support as 30 > BF > 100)*.* Transmission route Latin America to East Asia (BF = 4.11) and Western Africa to East Asia (BF = 3.48) was supported only in the WG model.

**Table 1.** Ranked Support Out of a Possible 56 Asymmetric Routes for VARV Transmission between Eight Discrete Regions from 1654 to 1977 by Modelled Loci Compared with the Whole Genome (WG).

Transmission Route		Data Set							
From	To	WG	*HA*	*ATI*	*CrmB*	*HA+ATI*	*HA+CrmB*	*ATI+CrmB*	*HA+ATI+CrmB*
Southern Asia	East Africa/Middle East	**1**	**2**	**2**	**10**	**1**	**8**	**10**	**2**
East Asia	Southern Asia	**2**	**1**	**12**	**6**	**2**	**2**	**9**	**4**
Southern Asia	Europe	**3**	**6**	**5**	**4**	**6**	**5**	**6**	**5**
Europe	Latin America	**4**	**3**	**1**	**7**	**3**	**4**	**4**	**3**
East Africa/Middle East	Southern Africa	**5**	**10**	**13**	**1**	**9**	**1**	**1**	**1**
Europe	Western Africa	**6**	**7**	**7**	**8**	**7**	**9**	**8**	**7**
Southern Asia	Asia Pacific	**7**	31	17	**3**	50	**7**	**3**	**8**
Latin America	East Asia	**8**	17	27	23	23	18	29	26
Europe	East Asia	**9**	**4**	**3**	**5**	**4**	**6**	**5**	**6**
Western Africa	East Asia	**10**	20	24	21	22	19	22	21

Note.—Supported routes in bold (Bayes Factor >3).

WG, whole genome; *HA*, hemagglutinin; *ATI*, A-type inclusion protein; *CrmB*, cytokine response modifier B.

In WG models supplemented with additional isolates sequenced for *HA* (WG + 20*HA*), some differences in transmission support can be observed when compared with WG models (table 2). Top supported routes Southern Asia to East Africa and the Middle East and East Asia to Southern Asia remained decisively supported (BF = 2207.14 and 118.42, respectively) across all data sets. Supported routes Latin America to East Asia (BF = 4.11) and Western Africa to East Asia (BF = 3.48) in the WG-only model were not significant (3 > BF) in the *HA* supplemented model (BF = 1.42 and 1.56 respectively). Supported route Europe to East Asia in the WG model increased to very strong support (100 > BF > 30), or from rank nine to rank three when supplemented with additional *HA* (BF = 4.07 to BF = 68.75 respectively). Route East Africa and Middle East to Southern Africa was reduced from rank five (BF = 82.31) to rank ten (BF = 5.29). Absolute Bayes Factor values for each route and model are available in supplementary tables S6 and S7, Supplementary Material online.

**Table 2.** Representativeness and Ranked Support Out of a Possible 56 Asymmetric Routes of VARV Transmission between Eight Discrete Regions by Taxon Data Set.

Transmission Route		Data Set		
From	To	WG	WG + 20*HA*	*HA *+* *20*HA*
Southern Asia	East Africa/Middle East	**1**	**1**	**2**
East Asia	Southern Asia	**2**	**2**	**1**
Southern Asia	Europe	**3**	**5**	**7**
Europe	Latin America	**4**	**4**	**3**
East Africa/Middle East	Southern Africa	**5**	**10**	**10**
Europe	Western Africa	**6**	**7**	**5**
Southern Asia	Asia Pacific	**7**	**6**	15
Latin America	East Asia	**8**	23	18
Europe	East Asia	**9**	**3**	**6**
Western Africa	East Asia	**10**	22	16

Note.—Supported routes in bold (Bayes Factor >3).

WG, whole genome taxonomic data set (*n* = 51); WG + 20*HA*, whole genome taxonomic data set (*n* = 51) supplemented with additional isolates sequenced for hemagglutinin only (*n* = 20); *HA *+* *20*HA*, hemagglutinin sequence extracted from whole genome isolates (*n* = 51) supplemented with additional isolates sequenced for hemagglutinin only (*n* = 20).

**Fig. 4.**
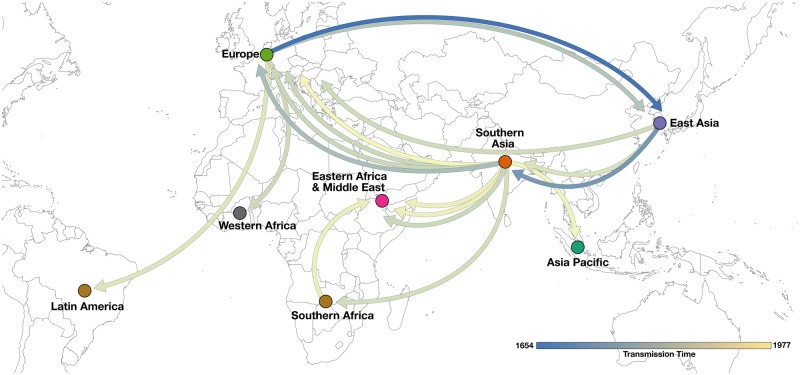
Projection between eight discrete regions using Bayesian phylogeography colored by sampling time (1654 and 1977) based on WG + 20*HA* taxon data set. Direction is indicated by arrows. Southern Asia is shown to be a modern hot spot for historic international transmission to Europe, Eastern Africa and the Middle East, Southern Africa and the Asia Pacific.

Figure 4 shows spatial projections of VARV based on the WG models supplemented with additional *HA* isolates (WG + 20HA). Considerable transmission originating from Southern Asia in the late 20th century can be observed. Consistent with our Bayes Factor analysis in table 2, Southern Asia represents one of only two discrete origin locations most frequently supported (*n* = 3), the second being Europe. Similarly, consistent Bayes Factor support for the wide dissemination of VARV from Europe to Latin America, East Asia, and Western Africa in table 2 can be observed in figure 4 with transmission occurring between the 17th and 20th centuries. Additional model parameters including molecular clock rates and divergence times are available as supplementary results, Supplementary Material online.

## Discussion

We have shown that VARV phylogenies based on reduced genetic data sets of *HA*, *ATI*, or *CrmB* can characterize historic VARV major isolates, and models combining *HA* and *ATI* or *HA*, *ATI*, and *CrmB* can further characterize minor subclades. Discrete-trait phylogeography has also empirically described historic routes of international smallpox transmission for the first time, and transmission events unresolved in models using currently available WG isolates are observed when supplemented with additional isolates sequenced for *HA* only. As the future promise of real-time phylogenetic analysis in the field supported by portable direct DNA sequencing technology begins to take shape (Faria et al. 2016; Gardy and Loman 2018), this is of diagnostic significance in the event of re-emergent smallpox as minimal genomic material could be promptly sequenced to support rapid outbreak response. These results likely owe to VARV’s low intergenomic sequence diversity (Esposito et al. 2006) meaning a high degree of precision is available to infer the relationship between epidemiologically linked isolates. Historic aggregate population CFR offer a reasonable but imperfect approximation of innate VARV pathogenesis by clade, as these estimates are known to be modified by the immunization status and specific age groups of affected persons (Esposito et al. 2006). However, as public health surveillance and epidemic field response are expected to increasingly rely on genomics in the future (Baele et al. 2016; Dellicour et al. 2017; Gardy and Loman 2018), understanding the potential for genes like *HA*, *ATI*, and *CrmB* to characterize VARV roughly according to pathogenesis could complement traditional laboratory methods (Loveless et al. 2009). Furthermore, an understanding of the relative advantages and limitations of phylogeography approaches to modelling VARV transmission is important should smallpox re-emerge.

The use of real-time phylogeography for outbreak response is an emerging concept but has yet to be applied during an epidemic. Whole genome Bayesian phylogeography of the 2014–2015 Ebola outbreak in West Africa revealed significant transmission events between contiguous Guinea, Liberia, and Sierra Leone that sustained the length of the epidemic (Dudas et al. 2017). It has been suggested that the real-time identification of these transmission chains could have improved response plans and potentially reduced the overall impact of the outbreak (Holmes et al. 2016). Others have used phylogeography to examine the emergence and spread of Zika virus across Africa and Asia (Faye et al. 2014). However, in time-sensitive outbreak settings, whole genomes may be unavailable due to limited sequencing capacity (Helmy et al. 2016). In the case of VARV, WG sequencing may also be impractical considering the size of the genome (Gilchrist et al. 2015), so rapidly sequencing diagnostic genes *HA, ATI*, and *CrmB* using standardized diagnostic protocols may provide a timely advantage for VARV characterization and the identification of transmission using phylogeography. Based on our results, models using *HA*, *ATI*, and *CrmB* could provide preliminary estimates of transmission to support response planning, however, decision makers should note the potential for reduced statistical support compared with WG models. Single gene models based on *HA*, *ATI*, or *CrmB* supported at least seven out of ten transmission routes supported in WG models from the same taxonomic data set, although rankings were discordant (table 1). Multilocus models combining *HA*, *ATI*, and *CrmB* genes support eight out of ten WG supported routes and discordance was reduced. Discordance between models however may be the result of recombination between VARV (Smithson, Kampman et al. 2014; Babkin and Babkina 2015), a disadvantage which could misinform response plans. In the event of recombination, multilocus models could therefore prove advantageous over WG models.

A combination of models using both WG and diagnostic genes such as *HA* like that shown in figure 3*B* could provide both timely information that reveals additional transmission events if the representativeness of the sample improves. In our study, when WG-only samples were supplemented with additional VARV taxa sequenced for *HA-*only, statistical support for transmission from Europe to East Asia increased from rank nine to rank three (table 2). This increase is likely due to the appearance of two separate clusters of East Asian/European taxa, one of which contains *HA-*only taxa (fig. 3*B* arrow). Of note, a taxon (AF377887/JPN44/HA) within this cluster is described as “Skin Lesion WWII” (supplementary table S2, Supplementary Material online). Historic reports describe the movement of smallpox from East Asia to Europe when infected soldiers were repatriated in 1944 following the end of WWII (Fenner et al. 1988). The appearance of these clusters provides some level of support (posterior probability 0.8) of at least two traceable lineages of smallpox circulating between East Asia and Europe following WWII. This approach of maximizing the sample size of phylogeography models by supplementing samples of WG-only taxa with isolates sequenced for *HA*-only may have improved the relative geographic and temporal representativeness of the sample, as these lineages were not observed in the smaller sample using isolates sequenced only for whole genomes (fig. 3*A*).

While this approach appears to resolve additional transmission events unseen in the WG-only models, the posterior probability on many other ancestral nodes within VARV Major in particular was reduced. This may be a critical limitation to the application of this approach, as even the most probable transmission events identified at some nodes may still be highly uncertain due to the introduction of large amounts of missing data into the alignment. The potential for misinformed estimates of transmission therefore increases with this uncertainty, meaning in some cases, determining the true origins of some transmission events may be difficult. In such instances, preference might be given to the Bayes factor analyses (table 2), which considers the statistical uncertainty of ancestral location states at each node, rather than limiting visualization to the most probable transmission events only (figs. 3 and 4). For example, we show that despite incongruent topologies, eight out of ten WG supported transmission routes were statistically supported in the supplemented model (WG + 20*HA*), and the top two were identically ranked (table 2), meaning sufficient data remained within nodes to identify these routes out of the possible 56. In practice, these results should ideally be interpreted in contrast to statistically stronger WG-only models. As outbreaks progress, preliminary models might be established using genes like as *HA, ATI*, and *CrmB*, and later replaced when whole genomes become available, therefore increasing certainty and supporting adaptive response planning. Studies examining the phylogeny of cowpox viruses however have shown distinct topologies can be generated from different genomic regions, sometimes with a high degrees of statistical support; another caveat to this approach (Dabrowski et al. 2013; Mauldin et al. 2017). This means that phylogeography models using *HA, ATI*, and *CrmB* could produce incorrect or discordant results (occasionally with high statistical support) relative to WG analyses. A degree of caution is therefore warranted if first interpreting the results of phylogeography based reduced data sets like *HA, ATI*, and *CrmB* without considering the potential for distinct topologies with high support.

The primary limitation of this study is the small sample size of VARV sequences available for analysis. Of the 571 VARV isolates currently stored in United States and the Russian Federation, 334 have date and location of sampling metadata (Alcami et al. 2010), meaning they could potentially be used for future phylogeography studies if made available. As additional VARV genomes have been uncovered in recent years outside of United States and Russian collections, the results of previous phylogenetic studies have been contested (Li et al. 2007; Duggan et al. 2016; Porter et al. 2017). We therefore cannot claim our results to be truly representative of past transmission when additional isolates remain unsequenced, only relative to the current gold-standard using all available WG taxa. Even if all additional isolates were sequenced and available for analysis, the sample could still be unrepresentative due to sampling bias. For example, the well-documented 1972 outbreak of VARV major in Yugoslavia following transmission from Iraq (Čobeljić 2004) is absent in figure 4. The strain circulating in Iraq at the time is believed to have been imported from Iran in 1971, itself imported from Afghanistan in 1970 (Tucker 2001). Figure 4 confirm this overall narrative, visualizing recent transmission from Southern Asia (Afghanistan) to Europe (Yugoslavia), however, as samples from Iraq are no longer stored or were never taken (Alcami et al. 2010), transmission via the Middle East will remain unresolved in any future phylogeography analysis. Another consequence of unrepresentative sampling can also be seen in figure 4, which shows transmission of minor Alastrim strains from Europe to Latin America in 1952. In this instance however, the reverse is true, as the European strain DQ441447/UNK52 is known to have been imported from Latin America (Fenner et al. 1988). Because isolates taken from Latin American prior to 1952 do not exist (Alcami et al. 2010), our analysis, and future analyses may still infer the direction incorrectly. Beside these notable cases, the sample is roughly consistent with the known spatial and temporal epidemiology of VARV at the time. In our study, just over half of all sequences (*n* = 39/71; 55%) were sampled between 1966 and 1980 during the WHO intensified smallpox eradication campaign when VARV circulation was most intense in Africa and Southern Asia (Fenner et al. 1988). Compared with other regions included in our analysis, a clear majority of sequences are sampled from Southern Asia and African and Middle Eastern countries (table 3), most of which were sampled after 1966, 86% (*n* = 13/19) and 73% (19/26) respectively. For sequences sampled prior to 1966, most (*n* = 19/33; 58%) come from Europe and East Asia when circulation and outbreaks were present, however other regions are still well represented.

**Table 3.** Count of Taxa Included for Analysis by Aggregate Region and Time Period.

Region	Count (WG)			Count (HA)			Total
	Total	Before 1966	After 1966	Total	Before 1966	After 1966	
Asia Pacific	2	0	2	0	0	0	2
Eastern Asia	5	5	0	4	4	0	9
East Africa and Middle East	11	3	8	2	1	1	13
Europe	9	8	1	2	2	0	11
Latin America	3	0	3	1	0	1	4
Southern Africa	6	2	4	1	0	1	7
Southern Asia	11	4	7	8	2	6	19
Western Africa	4	0	4	2	1	1	6
**Total**	51	22	29	20	10	10	71

Finally, the generalizability of our approach to other emerging VARV specific diagnostic targets such as *14 kDa* (Olson et al. 2004), *39 kDa*, and *A36R* (Kondas et al. 2015) is an important consideration. Sequence data sets analyzed under time-measured Bayesian phylogenetic assumptions such as phylogeography must contain a measurable accumulation of mutations over time, that is, a temporal signal (Baele et al. 2016). This is necessary to infer the relative similarity of related VARV sequences and subsequent associations to epidemiological processes such as location data. Sequences such as *14 kDa*, *39 kDa*, and *A36R* however are remarkably conserved (Olson et al. 2004; Kondas et al. 2015), such that the ability to differentiate between unrelated taxa sampled over time is difficult. As such, the generalizability of this approach is limited to targets that can demonstrate sufficient temporal signal. Considering this and the potential for recombination within OPV species (Smithson, Kampman et al. 2014; Babkin and Babkina 2015), future studies could investigate the utility of other diagnostic genes in both single and multilocus combinations with *HA*, *ATI*, and *CrmB* for characterization and phylogeography.

Overall, our results utilize the most comprehensive taxon data set publically available to date to explore VARV phylogeny, and the first to statistically model historic spread using Bayesian phylogeography. The methods used here have demonstrated potential utility for the rapid characterization of VARV strains based on diagnostic genes *HA*, *ATI*, and *CrmB*, as well as the exploration of VARV transmission which may be of use in outbreak situations should VARV re-emerge in the future.

## Conclusion

Our results show Bayesian multilocus phylogenetic models combining VARV genes *HA*, *ATI*, and *Crmb* can differentiate between VARV major and minor subclades roughly associated with high, intermediate and low CFR suggesting they may be used as indicators of potential mortality in future outbreaks. Multilocus Phylogeography models combining *HA*, *ATI*, and *CrmB* discordantly supported eight out of ten transmission routes supported by WG models and might be considered preliminary to inform outbreak response plans when whole genomes are unavailable. Increasing sample representativeness by supplementing WG phylogeography models of VARV with taxa sequenced for *HA* only could also be used to resolve additional transmission events compared with WG models alone, however, with increasing uncertainty. The methods used here are also the first to empirically describe global transmission of historic VARV isolates using phylogeography revealing two discrete lineages of VARV Major circulating between East Asia and Europe following WWII. Understanding phylogenetic and phylogeographic approach strategies for the rapid characterization of VARV is critical to support future preparedness planning and public health policy for epidemic response should smallpox re-emerge in the future.

## Materials and Methods

### Compilation of Sequence Data Sets

We performed a comprehensive search for all publicly available VARV gene and genome sequences. Of the 571 VARV samples remaining in United States and Russia, 48 whole genome (WG) isolates have been sequenced and are available via GenBank. Recently, three additional VARV isolates have been uncovered from historic remains and their genomes sequenced (Duggan et al. 2016; Pajer et al. 2017; Porter et al. 2017) totalling 51 WG isolates (supplementary table S1, Supplementary Material online). We identified 53 GenBank records for VARV isolates sequenced for *HA*. Cross referencing these 53 *HA* taxa to the 51 WG taxa with isolate records (Alcami et al. 2010) indicated that 31 *HA* taxa had duplicate sample sources with WG taxa, leaving 22 additional samples sequenced for *HA* but not yet as whole genomes. In order to maintain direct comparison between locations sampled between phylogeography models, two *HA* isolates, AF377891 and AF377893, sampled from United States in 1927 and 1940, were excluded from the final analysis. This has the effect of concealing transmission to and from United States as no other isolates from United States have been made publicly available. Table 3 details the count of the final data set by region and period. Additional details of *HA* taxa exclusion can be seen in supplementary table S2, Supplementary Material online, and breakdown of the final data set by country and aggregated regions can be seen in the supplementary table S3, Supplementary Material online.

Two of the three additional WG isolates (LT706528 and LT706529) discovered in historic remains come from the Czech Republic, and the other from Lithuania (dated prior to the divergence between major and minor VARV clades). Medical records associated with these isolates were destroyed during World War II meaning it is not possible to know the cause of death or condition of each respective case (Smrčka et al.; Pajer et al. 2017). Images present both specimens with discrete umbilicated pock lesions which are typical of ordinary and modified VARV major but also minor (Pajer et al. 2017). Therefore, it is insufficient to classify either on this basis also. The results of Pajer et al. (2017) classify LT706528 (V563) as VARV major and LT706529 (V1588) as alastrim minor (our results also mirror this). It is stated that V1588 has mutation D1705N within gene *O1L*, while the VARV major allocated V563 does not (Pajer et al. 2017). This gene has been investigated as a biomarker for VARV pathogenesis (Smithson, Purdy et al. 2014) and considering its basal position within the alastrim minor clade, this strain may have seeded Western African strains which later were imported into South America (De Jong 1956; Angulo 1976; Fenner et al. 1988; Li et al. 2007; Pajer et al. 2017).

### Phylogenetic Characterization and Phylogeography of Variola Virus

We used a Bayesian Markov Chain Monte Carlo procedure to characterize the phylogeny of the 51 heterochronously sampled VARV WG sequences as implemented in BEAST 1.8.4 (Drummond and Rambaut 2007; Ayres et al. 2012). We extracted sequences coding for diagnostics genes *HA, ATI*, and *CrmB* from WG VARV isolates using Geneious v10.1.2 (Kearse et al. 2012). Alignments were performed using MAFFT v7.3 (Katoh and Standley 2013) and visually inspected for errors. We used TempEst v1.5.1 (Rambaut et al. 2016) to demonstrate the suitability of each gene and gene combination for temporal analysis by a root-to-tip regression of genetic distance against sampling year (supplementary tables S3 and S4, Supplementary Material online). For this, maximum likelihood (ML) trees were generated using RAxML v8.2 (Stamatakis 2014). Where available, exact dates of sample isolation was used (Alcami et al. 2010). We set the sampling year for the three WG isolates sampled from historic remains (BK010317, LT706528, and LT706529) to 1654, 1929, and 1925 respectively based on previous work (Porter et al. 2017). We specified a general-time-reversible substitution model with invariant sites and site rate heterogeneity modelled across four gamma distributions (GTR+I + Γ_4_). We compared these preliminary models using path sampling and stone stepping marginal likelihood estimation (Baele, Lemey, et al. 2012; Baele, Li, et al. 2012). For final models, we specified the same substitution model under a strict molecular clocks and a constant coalescent tree prior based on model testing and previously published results (Duggan et al. 2016; Porter et al. 2017). We characterized sequences of *HA, ATI*, and *CrmB* independently and in multilocus partition models in various combinations to allow for independent rates of evolution across genes. We inspected log files for convergence and sufficient mixing using Tracer v1.6 (Rambaut et al. 2013) after removing 10% burn-in. We produced maximum clade credibility (MCC) trees using TreeAnnotator (Drummond and Rambaut 2007). We visually inspected trees for incongruence using FigTree v1.4.2 (Rambaut 2014).

For the phylogeographic analysis, we used BEAUti (Drummond and Rambaut 2007) to additionally specify an asymmetric discrete trait phylogeographic model utilizing a Bayesian stochastic search variable selection (BSSVS) framework (Lemey et al. 2009) as a metric for comparing geographic signal between data sets. For computational efficiency, we aggregated the 32 discrete sampling countries into eight discrete regions in the asymmetric model as per supplementary table S3, Supplementary Material online. We calculated Bayes factors indicating transmission support using SpreaD3 v0.9.6 (Bielejec et al. 2016). Support was defined as Bayes factor >3 as per convention (Lemey et al. 2009) with higher support interpreted according to supplementary table S8, Supplementary Material online (Jeffreys 1961). We generated phylogenetic plots using the ggtree library in R (Yu et al. 2017). For WG data sets supplemented with additional *HA* isolates, we specified the same Bayesian discrete trait models in BEAUTi as previously described to further delineate the phylogeography and investigate representativeness compared with the WG-only models. As mentioned above, all but two of the additional *HA* sequences fell within the eight discrete regions previously specified and were therefore removed to maintain comparison between models. Additional details of the methods are described in the Supplementary Material online.

## Supplementary Material

Supplementary Data
