# Supplementary material for: Bayesian Phylogeography and Pathogenic Characterization of Smallpox Based on HA, ATI, and CrmB Genes
Source: Mol Biol Evol. 2018 Aug 7;35(11):2607–17. doi: 10.1093/molbev/msy153 (PMC6231489; doi:10.1093/molbev/msy153)
Supplement: Supplementary Data [file msy153_supp.pdf]

Supplementary information:

Sequence Datasets

We downloaded all WG VARV sequences available from GenBank (n=54). Duplicate entries containing reassembled or re-sequenced isolates were removed leaving 51 unique WG taxa for analysis. Accession numbers for WG taxa are listed in Table S1 and duplicates removed listed in footnotes. We extracted gene regions *HA*, *ATI* and *CrmB* using annotations in Geneious 10.1.2 (Kearse et al. 2012) . We then downloaded all VARV isolates sequenced for *HA* available on GenBank to investigate representative sampling bias using *HA* (n=52) and to further delineate VARV phylogeography. We cross-referenced these *HA* isolates by name, year of sampling and location with the 51 WG isolates to identify unique isolates sequenced and uploaded to GenBank only for *HA* (Table S2) leaving 22 additional taxa for analysis. Year and location of sampling was extracted from each isolate from linked metadata or from published records (Organization 2010) by matching accession numbers or sequence names. Where available, exact dates of isolation was used. For sequences recently isolated from historic remains (BK010317, LT706528, LT706529) we specified sampling dates based on published analyses using molecular clocks (Duggan et al. 2016; Porter et al. 2017). Each sequence location was then aggregated into one of nine discrete regions: North America, Latin America, Europe, Western Africa, Eastern Africa & the Middle East, Southern Africa, Southern Asia, Asia Pacific, and Eastern Asia. No WG isolates and only two HA isolates were aggregated to North America and were removed to maintain comparison between all model leaving eight regions for analysis. Final sequences were aligned using MAFFT v7.3 (Katoh and Standley 2013).

Temporal Signal:

We confirmed the strong temporal signal of WG VARV (r2 = 0.714) as previously shown by Duggan et al (14) by a root-to-tip regression of genetic distance against year of sampling using the TempEst program (Rambaut et al. 2016). We analysed individual genes *HA*, *ATI*, and *CrmB* individually and concatenated in combination. Each gene and combination demonstrated reduced yet sufficient temporal signal for dated phylogenetic analysis under molecular clock assumptions (Table S3). For this maximum likelihood trees were generated for each set of taxa using RAxML (Stamatakis 2014) specifying a GTR nucleotide substitution model with 1000 bootstrap replicates. Each regression was plotted with and without sample sample BK010317 dated 1654 ACE to exclude the possibility of temporal bias introduced by the early sampling date (Table S4).

BEAST Model specification

We specified discrete trait phylogeography models using as BEAUti as part of the BEAST 1.8.4 package (Drummond and Rambaut 2007). Tip dates were imported with variable precision. For BK010317, LT706528 and LT706529, we set precision to 22.0, 15.0 and 16.0 years respectively around their estimated sampling date as inferred via their published methods (Duggan, et al. 2016; Porter, et al. 2017). For preliminary model testing we performed marginal likelihood calculations using a path sampling/stone stepping analysis with 50 steps (Baele et al. 2012; Baele et al. 2012). Models testing showed strict clock priors were preferable for VARV over relaxed clock models which also showed low rate heterogeneity across branches in agreement with the previously published methods (Duggan, et al. 2016; Porter, et al. 2017). Low posterior sampling (ESS) among early models with Skygrid tree priors also suggested insufficient data resulting in over-parametrization and lose

of statistical certainty so constant tree priors and strict molecular clocks were chosen for final analyses phylogeography analysis. We utilized a GTR+I+Γ_4 substitution model for WG models including WG models supplemented with *HA*. Final WG models were run for 100 million Markov Chain Monte Carlo generations (MCMC) in BEAST 1.8.4 and checked for convergence and mixing in Tracer 1.6 (Rambaut et al. 2014). For analysis of diagnostic genes *HA*, *ATI* and *CrmB* extracted from WG sequences, we specified single and multi-locus partition models using a simplified GTR+ Γ_4 substitution model due to insufficient posterior sampling when using a GTR+I+ Γ_4 substitution model which indicated over-parametrization of the reduced nucleotide data. These models were run for 20 million generations each also with strict clock and constant coalescent tree priors. For phylogeography in all models we specified an asymmetric transmission network between eight discrete regions selecting a Bayesian Stochastic Search Variable Selection Procedure (BSSVS) available in BEAST 1.8.4 (Drummond and Rambaut 2007). We generated Maximum Clade Credibility (MCC) trees from each group's posterior tree set using TreeAnnotator and after removing 10% of tree samples for burn-in. We obtained coordinates for each region using Google Earth by calculating the weighted average of taxa's longitude and latitude by sampling country. Statistical support for transmission routes were calculated using Spread3 0.9.7 (Bielejec et al. 2016). Statistically supported routed were specified as Bayes factor values greater than three. We visually inspected trees for incongruence using FigTree v1.4.2 (Rambaut 2009).

We estimated a mean genome-wide evolutionary rate of 1.20×10^{-5} substitutions per site per year, s/s/y (95% Bayesian credible interval = $1.12–1.28 \times 10^{-5}$ s/s/y). The mean rate of *HA*, *ATI*, and *CrmB* was 2.35×10^{-5}, 1.30×10^{-5} and 2.42×10^{-5} s/s/y respectively. For single gene and multi-locus models, the time to most recent common ancestor (tMRCA) had significantly wider credible intervals (range 324 years to 414 years, median 334 -345 years) compared to the WG (range 324 years to 335 years, median 328). Tip date sampling refined the estimated age of isolate BK010317 from 1654 to 1669 (95% Bayesian credible interval = 1658 – 1676) with the addition of six whole genomes and exact dates of sampling where available not previously used for analysis in published results.

Table S1: Compiled records of VARV isolates sequenced as full genomes and used for Bayesian phylogeography.

	**Accession #**	**Isolate name**	**Date isolated (yyyy-mm-dd)**	**Geographic origin**	**Repository description**
1	DQ437580	AFG70_vlt4	1970-03-18	Afghanistan	Variolator-4
2	DQ437581[a]	BSH75_banu	1975-11-24	Bangladesh	V75-550
3	DQ437582	CHN48_horn	1948	China	China Horn Sabin lab
4	DQ437583	CNG70_46	1970	Congo region	V70-46 Kinshasa
5	DQ437584	GER58_hdlg	1958	Germany	Heidelberg from India
6	DQ437585	IND64_vel4	1964	India	7124 Vellore
7	DQ437586	IND64_vel5	1964	India	7125 Vellore
8	DQ437587	IRN72_tbrz	1972	Iran	Iran 2602 Tabriz
9	DQ437588	NEP73_175	1973-07-26	Nepal	V73-175
10	DQ437589	PAK69_lah	1969-03-03	Pakistan	Rafiq Lahore
11	DQ437590	SOM77_ali	1977-11-10	Somalia	V77-2479 last case
12	DQ437591	SUM70_222	1970-10-17	Sumatra	V70-222
13	DQ437592	SYR72_119	1972-04-06	Syria	V72-119
14	DQ441416	BEN68_59	1968-04-10	Benin	V68-59, Dahomey
15	DQ441417	BOT72_143	1972-04-26	Botswana	V72-143
16	DQ441418	BOT73_225	1973-10-08	Botswana	V73-225
17	DQ441419	BRZ66_39	1966-06-05	Brazil	V66-39 alastrim
18	DQ441420	BSH74_nur	1974	Bangladesh	Nur Islam
19	DQ441421	BSH74_shz	1974	Bangladesh	Shahzaman
20	DQ441422	BSH74_sol	1974	Bangladesh	Solaiman
21	DQ441423	CNG70_227	1970-03-12	Congo region	V74-227 Gispen Congo 9
22	DQ441424	ETH72_16	1972-08-29	Ethiopia	Eth16 Addis
23	DQ441425	ETH72_17	1972-08-29	Ethiopia	ETH72_17 Eth17 Addis
24	DQ441426	GUI69_005	1969	Guinea	V69-005 Guinea
25	DQ441427	IND53_mad	1953-09-06	India	Kali-Muthu-Madras
26	DQ441428	IND53_ndel	1953	India	New Delhi
27	DQ441429	JAP46_yam	1946	Japan	Yamada MS-2A Tokyo

28	DQ441430	JAP51_hrpr	1951	Japan	Harper Masterseed
29	DQ441431	JAP51_stwl	1951	Japan	Stillwell Masterseed
30	DQ441432	KOR47_lee	1947	Korea	Lee Masterseed
31	DQ441433	KUW67_1629	1967-05-07	Kuwait	K1629
32	DQ441434	NIG69_001	1969	Nigeria	import from Nigeria
33	DQ441435	SAF65_102	1965-04-12	South Africa	102 Natal, Ingwavuma
34	DQ441436	SAF65_103	1965-04-14	South Africa	103 T'vaal, Nelspruit
35	DQ441437	SLN68_258	1968-01-02	Sierra Leone	V68-258
36	DQ441438	SOM77_1252	1977-05-19	Somalia	V77-1252
37	DQ441439	SOM77_1605	1977-08-09	Somalia	V77-1605
38	DQ441440	SUD47_jub	1947-10-07	Sudan	Juba (alastrim-like phenotype)
39	DQ441441	SUD47_rum	1947	Sudan	Rumbec
40	DQ441442	SUM70_228	1970-10-26	Sumatra	V70-228
41	DQ441443	TAN65_kem	1965-09	Tanzania	Kembula
42	DQ441444	UNK44_harv	1944	United Kingdom	Harvey Middlesex
43	DQ441445	UNK46_hind	1946	United Kingdom	Hinden
44	DQ441446	UNK47_hig	1947-04-06	United Kingdom	Higgins Staffordshire
45	DQ441447	UNK52_but	1952	United Kingdom	Butler alastrim
46	DQ441448	YUG72_164	1972	Yugosalvia	Yugoslavia from Iraq
47	X69198[b]	IND67_mah	1967	Brazil	Vector Maharastra E6
48	Y16780	BRZ66_gar	1966	Brazil	Garcia alastrim
	**Accession #**	**Isolate name**	**Year isolated**	**Geographic origin**	**Repository description**
1	BK010317[c]	VD21	1654	Lithuania	KY358055
2	LT706528	V563	1925	Czech	N/A
3	LT706529	V1588	1929	Czech	N/A

[a]DQ437581 is a resequence of CDC sample V75-550 (accession L22579 not listed).

[b] sequenced reference strain NC_001611 (not listed) is derived from accession X69196 (Esposito et al. 2006).

[c]KY358055 (VD21) from (Smithson et al. 2017) was recently reassembled and uploaded as BK010317.

Table S2: Compiled data of VARV isolates sequenced for HA and uploaded to GenBank. Isolates are cross referenced with WG VARV used for phylogeography. Likely unique and unique isolates (n=22) are used for analyses. Likely unique isolates share country and year of sampling with other WG isolates but have unique isolate names.

	**GeneBank accession**	**VARV isolate name**	**Year isolated**	**Geographic origin**	**Match to WG accession**
1	AY944055	BEN68_59	1968	Benin	DQ441416
2	AY944056	GUI69_005	1969	Guinea	DQ441426
3	AY944057	UNK52_butler	1952	United Kingdom	DQ441447
4	AY944058	BRZ66_39	1966	Brazil	DQ441419
5	AY944033	NEP73_175	1973	Nepal	DQ437588
6	AY944034	JAP46_yamada	1946	Japan	DQ441429
7	AY944035	JAP51_harper	1951	Japan	DQ441430
8	AY944036	IND53_New_Delhi	1953	India	DQ441428
9	AY944037	JAP51_stillwl	1951	Japan	DQ441431
10	AY944038	SAF65_102	1965	South Africa	DQ441435
11	AY944039	GER_heidel	1958	Germany	DQ437584
12	AY944040	UNK47_higgins	1947	United Kingdom	DQ441446
13	AY944041	UNK46_hinden	1946	United Kingdom	DQ441445
14	AY944042	CHN48_horn	1948	China	DQ437582
15	AY944043	IND53_mad	1953	India	DQ441427
16	AY944044	TAN65_kem	1965	Tanzania	DQ441443
17	AY944045	KOR47_lee	1947	Korea	DQ441432
18	AY944046	SUD47_jub	1947	Sudan	DQ441440
19	AY944047	SUM70_222	1970	Indonesia	DQ437591
20	AY944048	AFG70_vlt4	1970	Afghanistan	DQ437580
21	AY944049	IND64_vel4/7124	1964	India	DQ437585
22	AY944050	IRN72_tbrz	1972	Iran	DQ437587
23	AY944051	KUW67_1629	1967	Kuwait	DQ441433
24	AY944052	PAK69_lah	1696	Pakistan	DQ437589
25	AY944053	SYR72_119	1972	Syria	DQ437592

26	AY944054	YUG72_164	1972	Yugoslavia	DQ441448
27	AF375129	var-but	1952	United Kingdom	DQ441447
28	AF375141	var-raf	1969	Pakistan	DQ437589
29	AF375132	var-et16	1972	Ethiopia	DQ441424
30	AF375133	var-et17	1972	Ethiopia	DQ441425
31	AF375139	var-nur	1974	Bangladesh	DQ441420
32	AF375125	var-aba	1974	Bangladesh	Likely unique
33	AF375131	car-cng	1970	Congo	Likely unique
34	AF375134	var-hawa	1974	Bangladesh	Likely unique
35	AF375135	var-ilm	1965	Tanzania	Likely unique
36	AF375136	var-jal	1974	Bangladesh	Likely unique
37	AF375140	var-par	1974	Bangladesh	Likely unique
38	AF375143	var-som	1977	Somalia	Likely unique
39	AF375144	var-tlv	1946	United Kingdom	Likely unique
40	AF375130	var-cm6	1968	Brazil	Unique isolate
41	AF375138	var-nig	1961	Nigeria	Unique isolate
42	AF375142	var-sln	1968	Sierra Leone	Unique isolate
43	AF375126	var-af2	1972	Afghanistan	Unique isolate
44	AF375127	var-af3	1971	Afghanistan	Unique isolate
45	AF375128	var-bom	1958	India	Unique isolate
46	AF375137	var-mad	1962	India	Unique isolate
47	AF377887	Skin Lesion WWII	1944	Japan	Unique isolate
48	AF377888	Skin Lesion	1946	Japan	Unique isolate
49	AF377889	Skin Lesion	1945	China	Unique isolate
50	AF377890	Skin Lesion	1947	Belgium	Unique isolate
51	AF377891	Skin Lesion	1927	USA[a]	Unique isolate
52	AF377892	Skin Lesion	1946	Korea	Unique isolate
53	AF377893	Skin Lesion	1940	USA[a]	Unique isolate

[a]Unique but not included in final analyses to maintain comparison with discrete locations in WG models

Table S3: Summary of geographic location of unique isolates by country, aggregate region and genetic content.

Region & Country of isolation	WG	HA	Total
**Asia Pacific**	**2**	**0**	**2**
Indonesia	2	0	2
**Eastern Asia**	**5**	**4**	**9**
Japan	3	2	5
South Korea	1	1	2
China	1	1	2
**East Africa & Middle East**	**11**	**2**	**13**
Kuwait	1	0	1
Iran	1	0	1
Syria	1	0	1
Somalia	3	1	4
Ethiopia	2	0	2
Sudan	2	0	2
Tanzania	1	1	2
**Europe**	**9**	**2**	**11**
Belgium	0	1	1
United Kingdom	4	1	5
Yugoslavia	1	0	1
Germany	1	0	1
**Latin America**	**3**	**1**	**4**
Brazil	3	1	4
**Southern Africa**	**6**	**1**	**7**
Congo	2	1	3
South Africa	2	0	2
Botswana	2	0	2
**Southern Asia**	**11**	**8**	**19**
Afghanistan	1	2	3
Bangladesh	4	4	8
Nepal	1	0	1
India	4	2	6
Pakistan	1	0	1
**Western Africa**	**4**	**2**	**6**
Nigeria	1	1	2
Sierra Leone	1	1	2
Guinea	1	0	1
Benin	1	0	1
**Total**	**51**	**20**	**71**

Table S4: Summary of root-to-tip genetic distance by sampling year of VARV (51 Taxa)

Statistic	WG	HA	ATI	crmB	HA+ATI	HA+crmB	ATI+crmB	HA+ATI+crmb
Slope (rate)	1.18E-05	1.19E-05	1.16E-05	2.28E-05	1.17E-05	1.75E-05	1.67E-05	1.50E-05
X-Intercept	1660.5226	1691.4635	1647.296	1662.0872	1664.769	1671.623	1649.352	1663.6102
Correlation Coefficient	0.8451	0.4055	0.7979	0.6835	0.6697	0.749	0.8584	0.8204
R squared	0.7143	0.1644	0.6367	0.4672	0.4485	0.561	0.7368	0.673
Residual Mean Squared	1.15E-07	1.48E-06	1.58E-07	1.22E-06	3.46E-07	4.94E-07	2.07E-07	2.25E-07

Table S5: Summary of root-to-tip genetic distance by sampling year of VARV after removing BK010317 (50 Taxon)

Statistic	WG	HA	ATI	crmB	HA+ATI	HA+crmB	ATI+crmB	HA+ATI+crmb
Slope (rate)	1.42E-05	4.26E-05	1.41E-05	3.07E-05	2.51E-05	2.29E-05	1.96E-05	2.59E-05
X-Intercept	1833.1281	1871.4638	1847.7916	1849.0064	1863.555	1840.494	1816.926	1841.7691
Correlation Coefficient	0.4907	0.5062	0.3772	0.2152	0.5222	0.4313	0.3218	0.4556
R squared	0.2408	0.2562	0.1422	4.63E-02	0.2727	0.186	0.1035	0.2075
Residual Mean Squared	1.01E-07	8.42E-07	1.92E-07	3.09E-06	2.69E-07	3.67E-07	5.32E-07	4.08E-07

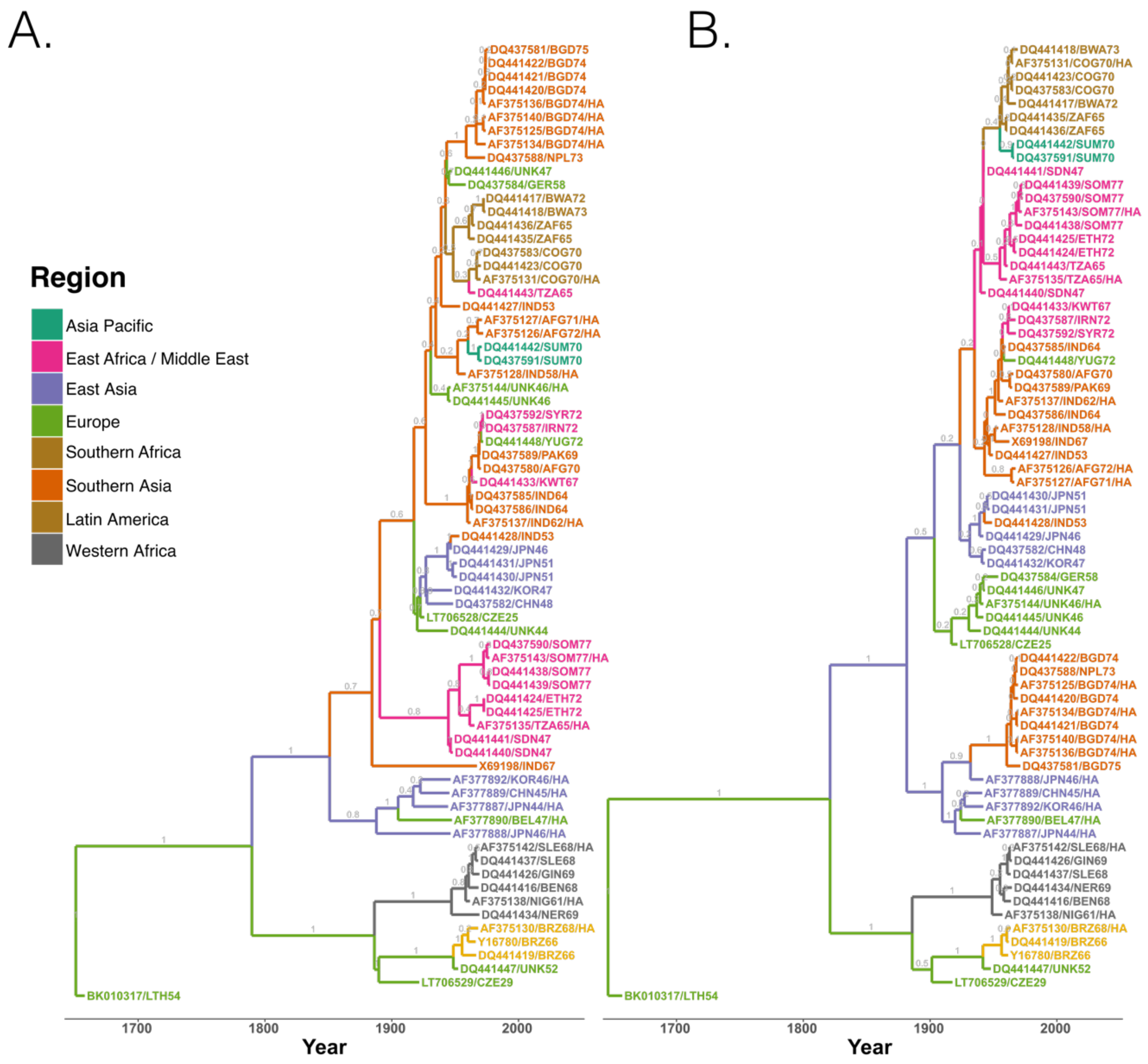

Figure S1: Time-rooted phylogenetic characterisation of 71 VARV isolates. Values on ancestral nodes represent posterior probabilities. Tip names are coloured by sampling region and edges coloured by inferred origin using a BSSVS framework. **A**: Fifty-one whole genome VARV isolates aligned with 20 additional *ha* sequences. **B**: Fifty-one *ha* sequences extracted from whole genome VARV isolates aligned with 20 additional *ha* sequences.

Table S6. Absolute Bayes Factor support for VARV transmission between eight discrete regions from 1654 to 1977 by dataset using Bayesian phylogeography. Supported routes in bold (Bayes Factor >3)

**Transmission Route**		**Dataset**							
From	To	WG	*HA*	*ATI*	*CrmB*	*HA+ATI*	*HA+CrmB*	*ATI+CrmB*	*HA+ATI+CrmB*
Southern Asia	East Africa/Middle East	**4703.65**	**50.18**	**22.21**	**5.19**	**39.63**	**7.68**	**5.35**	**61.65**
East Asia	Southern Asia	**542.45**	**90.83**	**3.55**	**11.11**	**34.92**	**92.01**	**5.36**	**24.17**
Southern Asia	Europe	**330.14**	**9.12**	**7.28**	**22.69**	**7.88**	**24.54**	**12.95**	**19.21**
Europe	Latin America	**107.21**	**39.04**	**32.83**	**8.72**	**33.36**	**25.96**	**21.19**	**34.06**
East Africa/Middle East	Southern Africa	**82.31**	**3.48**	**3.50**	**397.44**	**5.99**	**302.57**	**340.42**	**320.42**
Europe	Western Africa	**11.84**	**8.59**	**6.09**	**6.52**	**7.33**	**7.41**	**5.72**	**7.59**
Southern Asia	Asia Pacific	**8.37**	1.27	2.52	**24.15**	0.96	**11.21**	**25.19**	**6.59**
Latin America	East Asia	**4.11**	1.81	1.46	1.51	1.51	1.68	1.43	1.53
Europe	East Asia	**4.07**	**18.24**	**13.09**	**18.66**	**16.09**	**21.17**	**16.61**	**12.37**
Western Africa	East Asia	**3.48**	1.68	1.53	1.53	1.52	1.64	1.58	1.57

Table S7. Representativeness by absolute support of VARV transmission between eight discrete regions from 1654 to 1977 by dataset using Bayesian phylogeography. Supported routes in bold (Bayes Factor >3)

**Transmission Route** From	To	**Dataset** WG	WG+20*HA*	*HA*+20*HA*
Southern Asia	East Africa/Middle East	**4703.65**	**2207.14**	**55.90**
East Asia	Southern Asia	**542.45**	**118.42**	**507.53**
Southern Asia	Europe	**330.14**	**38.44**	**10.06**
Europe	Latin America	**107.21**	**60.79**	**44.64**
East Africa/Middle East	Southern Africa	**82.31**	**5.29**	**4.18**
Europe	Western Africa	**11.84**	**9.45**	**5.00**
Southern Asia	Asia Pacific	**8.37**	**10.78**	2.25
Latin America	East Asia	**4.11**	1.42	2.10
Europe	East Asia	**4.07**	**68.75**	**10.50**
Western Africa	East Asia	**3.48**	1.56	2.25

Table S8. Interpretation of computed Bayes Factor values

Bayes Factor (BF)	Interpretation of Evidence
>100	Decisive
30-100	Very strong
10-30	Strong
3-10	Substantial

Adapted from (Liang and Xiong 2013) Table 1 and (Jeffreys 1961)

Supplementary References

Baele G, Lemey P, Bedford T, Rambaut A, Suchard MA, Alekseyenko AV. Improving the accuracy of demographic and molecular clock model comparison while accommodating phylogenetic uncertainty. Molecular biology and evolution 2012;29(9):2157-2167.

Baele G, Li WLS, Drummond AJ, Suchard MA, Lemey P. Accurate model selection of relaxed molecular clocks in Bayesian phylogenetics. Molecular biology and evolution 2012;30(2):239-243.

Bielejec F, Baele G, Vrancken B, Suchard MA, Rambaut A, Lemey P. Spread3: Interactive visualization of spatiotemporal history and trait evolutionary processes. Molecular biology and evolution 2016;33(8):2167-2169.

Drummond AJ, Rambaut A. BEAST: Bayesian evolutionary analysis by sampling trees. BMC evolutionary biology 2007;7(1):214.

Duggan AT, Perdomo MF, Piombino-Mascali D, Marciniak S, Poinar D, Emery MV, Buchmann JP, Duchêne S, Jankauskas R, Humphreys M. 17 th Century Variola Virus Reveals the Recent History of Smallpox. Current Biology 2016;26(24):3407-3412.

Esposito JJ, Sammons SA, Frace AM, Osborne JD, Olsen-Rasmussen M, Zhang M, Govil D, Damon IK, Kline R, Laker M. Genome sequence diversity and clues to the evolution of variola (smallpox) virus. Science 2006;313(5788):807-812.

Jeffreys H. The theory of probability. OUP Oxford; 1961.

Katoh K, Standley DM. MAFFT multiple sequence alignment software version 7: improvements in performance and usability. Molecular biology and evolution 2013;30(4):772-780.

Kearse M, Moir R, Wilson A, Stones-Havas S, Cheung M, Sturrock S, Buxton S, Cooper A, Markowitz S, Duran Cet al. Geneious Basic: An integrated and extendable desktop software platform for the organization and analysis of sequence data. Bioinformatics 2012;28(12):1647-1649.

Liang F, Xiong M. Bayesian Detection of Causal Rare Variants under Posterior Consistency. PLOS ONE 2013;8(7):e69633.

Organization WH. Scientific review of varíola vírus research, 1999-2010. 2010.

Porter AF, Duggan AT, Poinar HN, Holmes EC. Comment: Characterization of Two Historic Smallpox Specimens from a Czech Museum. Viruses 2017;9(10):276.

Rambaut A. FigTree. 1.4.22009.

Rambaut A, Lam TT, Max Carvalho L, Pybus OG. Exploring the temporal structure of heterochronous sequences using TempEst (formerly Path-O-Gen). Virus Evolution 2016;2(1):vew007.

Rambaut A, Suchard M, Xie D, Drummond A. Tracer v1.6 2014.

Smithson C, Imbery J, Upton C. Re-Assembly and Analysis of an Ancient Variola Virus Genome. Viruses 2017;9(9):253.

Stamatakis A. RAxML version 8: a tool for phylogenetic analysis and post-analysis of large phylogenies. Bioinformatics 2014;30(9):1312-1313.
